# Gut Associated Metabolites Enhance PD-L1 Blockade Efficacy in Prostate Cancer

**DOI:** 10.32604/or.2025.072661

**Published:** 2026-01-19

**Authors:** Ke Liu, Xia Xue, Haiming Qin, Jiaying Zhu, Meng Jin, Die Dai, Youcai Tang, Ihtisham Bukhari, Hangfan Liu, Chunjing Qiu, Feifei Ren, Pengyuan Zheng, Yang Mi, Weihua Chen

**Affiliations:** 1 Henan Key Laboratory for Helicobacter Pylori and Digestive Tract Microecology, The Fifth Affiliated Hospital of Zhengzhou University, Zhengzhou, 450001, China; 2 Institute of Rehabilitation Medicine, Henan Academy of Innovations in Medical Science, Zhengzhou, 450001, China; 3 Tianjian Laboratory of Advanced Biomedical Sciences, Zhengzhou University, Zhengzhou, 450001, China; 4 Biotherapy Center, The First Affiliated Hospital of Zhengzhou University, Zhengzhou, 450001, China; 5 School of Public Health, Zhengzhou University, Zhengzhou, 450001, China; 6 Key Laboratory of Molecular Biophysics of the Ministry of Education, Hubei Key Laboratory of Bioinformatics and Molecular-Imaging, Center for Artificial Intelligence Biology, Department of Bioinformatics and Systems Biology, College of Life Science and Technology, Huazhong University of Science and Technology, Wuhan, 430074, China; 7 School of Biological Science, Jining Medical University, Rizhao, 276800, China

**Keywords:** Gut microbiome, metabolites, prostate cancer, programmed death-ligand 1, immunotherapy, gut-tumor metabolic axis

## Abstract

**Background:**

The gut microbiome has emerged as a critical modulator of cancer immunotherapy response. However, the mechanisms by which gut-associated metabolites influence checkpoint blockade efficacy in prostate cancer (PC) remain not fully explored. The study aimed to explore how gut metabolites regulate death-ligand 1 (PD-L1) blockade via exosomes and boost immune checkpoint inhibitors (ICIs) in PC.

**Methods:**

We recruited 70 PC patients to set up into five subgroups. The integrated multi-omics analysis was performed. In parallel, we validated the function of gut microbiome-associated metabolites on PD-L1 production and immunotherapy treatment efficacy in PC cell lines and transgenic adenocarcinoma of the mouse prostate (TRAMP) models.

**Results:**

We identified two metabolites, 16(R)-Hydroxyeicosatetraenoic acid (16(R)-HETE) and 6-Keto-Prostaglandin E1 (6-Keto-PGE1), that positively correlated with the plasma exosomal PD-L1 levels. The *in vitro* experiments found that both 16(R)-HETE and 6-Keto-PGE1 can enhance PD-L1 expression at the mRNA, protein, and exosome levels in both human and mouse PC cell lines, which were also validated *in vivo* based on subcutaneous mouse models. Both metabolites significantly promoted the anti-PD-L1 efficacy against PC *in situ* on a TRAMP mouse model.

**Conclusions:**

Targeting the “gut-tumor metabolic axis” is a promising strategy to improve the efficacy of immune checkpoint inhibitors in tumors.

## 1 Introduction

The gut microbiome nowadays has been recognized as a key driver in modulating the efficacy of cancer immunotherapy response, with specific mechanisms potentially involving the activation of the Stimulator of Interferon Genes (STING)–type I interferon pathway and the γδ T cell–APC–CD8^+^ T cell axis [1–3]. Increasing evidence indicates that the complex interplay between the gut microbial ecology and the host immune system can significantly influence the efficacy of immune checkpoint inhibitors [4–6]. However, the mechanisms behind this, particularly in prostate cancer (PC) treatment, remain unclear. Among the most common cancers, PC is the second most frequent, and it is the fifth most common cause of cancer-related deaths worldwide [7,8]. According to the updated guideline and recent clinical trials, ICIs exhibit limited efficacy in PC [9]. While ICIs have been reported to be promising in part of PC individuals, the overall response rates remain around 30%, underestimating the need to better understand the variables that determine treatment response [10–12]. The important role that the gut microbiota and fecal metabolites have in influencing the tumor immune microenvironment and boosting the effectiveness of cancer immunotherapies have been brought to light by a number of studies regarding the interaction between the gut microbiome and cancer [13,14].

Although the primary PC can be controlled effectively through androgen deprivation therapy (ADT) [15–17], it is highly likely that PC patients develop resistance to ADT and eventually progress to castration-resistant prostate cancer (CRPC) and tumor metastasis [18]. Indeed, second-line therapy such as abiraterone and enzalutamide can prolong patients’ survival to a certain extent, but patients may still experience drug resistance [18–20]. It has been found that the status of gut microbes and metabolites can contribute to the extra-gastrointestinal carcinogenesis and therapy effectiveness, as well as drive host immunity and anti-Programmed Cell Death Protein 1/Programmed Cell Death Ligand 1 (PD1/PD-L1) treatment [21]. According to earlier research, PC patients had less of a variety of gut microbiota than paired-normal controls [22,23]. Briefly, pro-inflammatory *Bacteroides* and *Streptococcus* increased significantly in PC patients, while *Akkermansia muciniphila*, *Ruminococcaceae*, and *Lachnospiraceae* in the ADT group were more dominant than those of the untreated group [24]. In CRPC patients, *A*. *muciniphila* was enriched in intestinal flora after being treated with abiraterone acetate. In parallel, the level of vitamin K2, a metabolite with anti-tumor effects, was significantly increased in feces from PC patients [24,25]. The current studies on immune microbiota and PC are limited to microbiota investigation and correlation analysis; it is thus valuable to establish a systematic analysis of fecal metabolome and its associations with gut microbiota in PC to promote its diagnosis and treatments.

Notably, the cytosolic or plasma level of PD-L1 was significantly increased in PC cell lines as well as animal models, which suggests the possibility of combining anti-PD1/PD-L1 therapy with regular treatment in this type of tumor [26–28]. The gut microbiota may be used to modify the effectiveness of anti-PD1/PD-L1 therapy because the latter may have a worthexploring link (mechanisms and metabolomes) between PD-L1 and microbial changes in gut environments in PC patients. The discovery of PD-L1 expression on tumor-derived extracellular vesicles, particularly exosomes, has added another layer of complexity to the understanding of PD-L1-mediated immune regulation in the tumor microenvironment [29–31]. Exosomal PD-L1 is secreted by living cells and can reflect the circulating immune inhibition state, which is more convenient than *in situ* pathological biopsy [32,33]. It is an effective way to predict and observe immunotherapeutic effects that can be conveniently monitored during the course of treatment [33]. Exo-PD-L1 can cause T cell exhaustion and block the anti-tumor immune response, even in the absence of surface PD-L1 expression on the tumor cells themselves, by interacting with PD-1 on T cells [34,35]. Several studies have reported the presence of elevated exosomal PD-L1 in the plasma or serum from cancer patients compared with healthy individuals [36,37]. The levels of exo-PD-L1 have been associated with disease stage, metastatic status, and response to immune checkpoint inhibitor therapy [38–40]. However, there is a gap in understanding the precise mechanisms by which the gut microbiome and fecal metabolites influence the release and function of exosomal PD-L1 in PC.

In this study, we explored the alterations of the gut ecosystem in PC patients, particularly during cancer development and treatment, and investigated their relationships with exosomal PD-L1 levels. Based on multi-omics, we aimed to identify the interplay between gut microbe-derived metabolites and exosomal PD-L1 in PC. In parallel, this work was conducted both *in vitro* and *vivo* to validate the mechanistic insights into how these gut-microbiome-associated metabolites influenced PD-L1 expression and the efficacy of anti-PD-L1 therapy. Our study would reveal the “gut-tumor metabolic axis” and its impact on immunotherapy response, which could provide strategies for development to enhance the efficacy of cancer immunotherapies.

## 2 Materials and Methods

### 2.1 Patients Recruitment

A total of 70 Chinese patients (46 to 87 years old) who were diagnosed with PC at the Fifth Affiliated Hospital of Zhengzhou University during 2024–2025 were recruited for this investigation. An ethical approval was also obtained from the research ethics committee and review board of the Fifth Affiliated Hospital of Zhengzhou University (KY2024058-K02). It was noted that there was variation in the environment as well as in diet and genetics. Patients with a history of taking antibiotics for more than three months were excluded. In this study, patients who were identified as having benign prostatic hyperplasia were classified as controls. Notably, patients in the control group did not have prostate biopsies but had undergone complete hematologic tumor marker screening and imaging examination based on all negative reports; they were thus defined as benign tumors.

Recruited patients were divided into five groups: B (benign prostatic hyperplasia), N (no drugs), S (hormone-sensitive), R (castration-resistant), and AA (castration-resistant with abiraterone acetate). Patients in group N have not been treated with ADT, while those in group S were treated with gonadotropin-releasing hormone (GNRH) agonist/antagonist and oral androgen receptor axis-targeted (ATT) therapies before radical resection of PC or diagnosed with metastasis without radical prostatectomy. Patients in group R were resistant to primary androgen deprivation therapy, so they were treated with GNRH agonist/antagonist and oral ATT. The patients in the AA group were treated with GNRH agonist/antagonist and abiraterone acetate and were resistant to castration. The clinicopathological features of all patients enrolled in our study are provided in Table 1.

**Table 1:** Clinical characteristics of all subject cases (n = 70)

Characteristics	B (n = 8)	N (n = 14)	S (n = 28)	R (n = 13)	AA (n = 7)	*p* Value
Age (years)	65.8 ± 5.8	65.6 ± 9.7	69.5 ± 5.5	72.4 ± 7.1	65.3 ± 7.6	0.951
BMI (kg/m^2^)	25.1 (22.9–25.5)	24.2 (22.4–26.9)	24.4 (22.9–26.8)	24.8 (22.3–26.1)	23.7 (22.5–24.8)	0.932
PSA (ng/mL)	12.4 (8.4–20.3)	61.5 (18.9–215.1)	0.9 (0.2–4.6)	32.0 (16.1–77.6)	25.0 (4.4–109.0)	0.027^abcd^
Gleason Score	—	8.5 (7.0–9.0)	8.0 (7.0–9.0)	8.0 (7.5–9.0)	105.0 (55.0–124.0)	0.405
AKP (40–130 U/L)	75.5 (63.8–85.8)	64.5 (57.5–197.8)	70.5 (59.5–79.0)	70.0 (60.5–192.0)	105.0 (55.0–124.0)	0.903
Therapy	—	—	ADT	ADT	ADT + AA	
MD (month)	—	—	2.3 (1.0–5.8)	12.0 (6.2–36.0)	10.0 (4.0–12.0)	0.007^bcde^
Metastasis	—	n = 6	n = 10	n = 12	n = 6	0.074

Note: AKP: Alkaline Phosphatase; BMI: Body Mass Index; PSA: Prostate Specific Antigen; MD: Medication Duration; B: benign prostatic hyperplasia; N: no drugs; S: hormone-sensitive; R: castration-resistant; AA: castration-resistant with abiraterone acetate; ^a^*p* < 0.05 stands for B vs. N; ^b^*p* < 0.05 stands for N vs. S; ^c^*p* < 0.05 stands for N vs. R; ^d^*p* < 0.05 stands for S vs. R; ^e^*p* < 0.05 stands for R vs. AA.

### 2.2 Blood Sample Collection

All the participants were asked to fast overnight (10–12 h) before sampling. The following morning, peripheral blood specimens were collected and subjected to centrifugation at 1300× *g* for 10 min, followed by 3000× *g* for 15 min. Plasma samples were isolated and stored at −80°C for future use.

### 2.3 Microbiome Analysis

#### 2.3.1 Stool Sample Collection

Feces (middle and rear portion) were collected from all patients in the sterile feces collection containers at the urological ward, then immediately stored at −80°C until processed.

#### 2.3.2 DNA Extraction and PCR Amplification

Genomic DNA from microbes was extracted from fecal samples using magnetic beads, following the manufacturer’s instructions provided in the fecal genomic DNA extraction kit (TIANGEN, DP712, Beijing, China). 1% agarose gel electrophoresis was employed to assess the quality of the isolated DNA samples, while their concentration and purity were quantified using a NanoDrop 2000 UV-vis spectrophotometer (Thermo Scientific, Waltham, MA, USA). With the ABI GeneAmp^®^ 9700 PCR thermocycler (ABI, Foster City, CA, USA), the V3-V4 hypervariable region of bacterial 16S rRNA was amplified using the primer pairs 338F (5^′^-ACTCCTACGGGAGGCAGCAG-3^′^) and 806R (5^′^-GGACTACHVGGGTWTCTAAT-3^′^). The amplification protocol for the target sequence was as follows: an initial denaturation step at 95°C for 3 min, followed by 27 cycles consisting of denaturation at 95°C for 30 s, annealing at 55°C for 30 s, and extension at 72°C for 45 s, a single final extension at 72°C for 10 min, and a final hold at 4°C. Each 20 μL PCR reaction mixture contained 4 μL of 5 × TransStart FastPfu buffer, 2 μL of 2.5 mm dNTPs, 0.8 μL of forward primer (5 μm), 0.8 μL of reverse primer (5 μm), 0.4 μL of TransStart FastPfu DNA Polymerase, 10 ng of template DNA, and ddH_2_O added to reach the final volume. All PCR reactions were run in triplicate. PCR amplicons were excised from 2% agarose gels, purified with the AxyPrep DNA Gel Extraction Kit (Axygen Biosciences, 11696505001, Union City, CA, USA) following the manufacturer’s recommended procedures, and their concentrations were determined using a Quantus™ Fluorometer (Promega, E6150, Madison, WI, USA).

#### 2.3.3 Illumina MiSeq Sequencing

Purified amplicons were pooled in equimolar amounts and subjected to paired-end sequencing (2 × 300 bp) on an Illumina MiSeq platform (Illumina, San Diego, CA, USA). The sequencing library was prepared using the MiSeq Reagent Kit v3 (600-cycle) (Illumina, MS-102-3003, San Diego, CA, USA) following the standard protocols provided by Majorbio Bio-Pharm Technology Co., Ltd. (Shanghai, China).

#### 2.3.4 Processing of Sequencing Data

The 16S rRNA sequence data were filtered and analyzed using QIIME2 (version 2023.9.0) [41]. Sequencing errors and chimeric sequences were detected and denoised by the DADA2 algorithm embedded in QIIME2 [42], with parameters set to “–p-trim-left-f 0 –p-trim-left-r 0 –p-trunc-len-f 280 –p-trunc-len-r 220”. After sequence filtering, taxonomy was assigned to amplicon sequence variants (ASV) using Naïve Bayes classifiers trained on the V4 region of the “SILVA 138 SSURef NR99” dataset via the q2-feature-classifier plugin [43]. The ASV feature tables were aggregated to the genus level using q2-taxa and subsequently employed to determine the relative abundance for each sample. Genera exhibiting a maximum abundance greater than 0.001 and an average abundance greater than 0.0001 across all samples were retained for downstream analysis.

#### 2.3.5 Gas Chromatography-Mass Spectrometry (GC-MS) Analysis

The analysis was performed using an Agilent 8890B gas chromatograph coupled to an Agilent 5977B mass selective detector (Agilent Technologies, Santa Clara, CA, USA). This system featured an inert electron impact ionization (EI) source functioning at 70 eV (Agilent, USA). A HP-FFAP (30, 0.25 m), a capillary column, was used to separate the analyte compounds using 99.999% helium as a carrier gas at a constant flow rate (1 mL/min). The GC column temperature was held at 80°C and raised to 120°C with a rate of 40°C per minute, then raised to 200°C with a rate of 10°C per minute, finally held at 230°C for 6 min. A 1 µL of sample was injected in splitting mode (10:1) with the inlet temperature of 260°C. The ion source temperature was 230°C and the quadrupole temperature was 150°C. Data collection was conducted in full scan mode with a range of m/z 30–300. Compounds were identified and quantified using the MassHunter software (v10.0.707.0, Agilent, Santa Clara, CA, USA).

### 2.4 Untargeted Metabolomics Analysis

#### 2.4.1 Metabolite Extraction

The 50 mg solid samples of human feces were taken for the extraction of metabolites in a 400 µL methanol: water solution (4:1, v/v). The mixture was treated by high-throughput tissue crusher Wonbio-96c (Shanghai Wanbo Biotechnology Co., Ltd., China) at 50 Hz for six minutes, then followed by 30 s vortex and ultrasonography at 40 kHz for thirty minutes at 5°C after being allowed to settle at −20°C. The samples were stored at −20°C for 30 min to allow protein precipitation. After centrifuging at 13,000× *g* for 15 min (4°C), the supernatant was carefully transferred to sample vials for Liquid Chromatography-Tandem Mass Spectrometry (LC-MS/MS) analysis.

#### 2.4.2 Quality Control Sample

As a part of the system conditioning and quality control process, a pooled quality control (QC) sample was prepared by mixing equal volumes of all fecal metabolomic samples. The QC samples were disposed, and tested in the same manner as the analytic samples. It helped to represent the whole sample set, which would be injected at regular intervals (every 6–8 samples) to monitor the robustness of the analysis.

#### 2.4.3 LC-MS/MS Analysis

The samples (10 µL) were analyzed using a BEHC 18 column (75 µm × 25 cm, Thermo, USA). With a flow rate of 0.4 mL/min, the column temperature was kept at 40°C. The mobile phases were: Solvent B-0.1% acetonitrile/isopropanol and Solvent A-0.1% formic acid in Milli-Q water. The samples were analyzed using a Q-Exactive Focus mass spectrometer (Thermo) fitted with an electrospray ionization (ESI) source, with the heated capillary maintained at 500°C. The spray voltage was set to 5 kV in positive mode and 4 kV in negative mode. The mass spectrometer was operated in full scan mode (m/z 50–1000) for all exosome analyses, using both positive and negative ionization modes. Nitrogen was used as the sheath and auxiliary gases, set to 50 and 50 arbitrary units, respectively.

#### 2.4.4 Data Pre-Processing

The raw data were imported into the Progenesis QI metabolomics processing software (Waters Corporation, version 2.0, Milford, MA, USA). This workflow included baseline filtering, peak identification, integration, retention time correction, and peak alignment to generate a data matrix of retention time, mass-to-charge ratio, and peak intensity. The data were then pre-processed with the following steps: (1) Retained only variables present in at least 80% of samples in one group; (2) Filled in missing values using the minimum value from the original data matrix; (3) Normalized the total peak intensity across samples; (4) Removed variables with a relative standard deviation (RSD) ≥ 30% in the QC samples. Finally, to get the finished processed data matrix for downstream analysis, the data were log-transformed.

### 2.5 Differential Metabolite Identification and Enrichment Analysis

Differential metabolites were detected by conducting pairwise comparisons among the groups. For each comparison non-parametric Wilcoxon rank-sum test was done, and metabolites with *p* < 0.05 were considered significant. In parallel, partial least squares discriminant analysis (PLS-DA) was conducted using the plsda function in the ‘mixOmics’ package (version 6.32.0), and metabolites with a variable importance in projection (VIP) score greater than 1.0 were retained. The intersection of the two approaches was defined as the final set of differential metabolites. Analyses were performed separately for the positive and negative ionization modes, and the resulting metabolite lists were subsequently merged, yielding a total of 357 differential metabolites (Supplementary Table S1).

To assess the biological relevance of these metabolites, all compound–pathway associations were retrieved from the Kyoto Encyclopedia of Genes and Genomes (KEGG) database (https://www.kegg.jp/) and used as the background reference set (accessed on 08 July 2024). Enrichment analysis was carried out using the enrichr function in the ‘clusterProfiler’ package (version 4.16.0), applying a hypergeometric test to identify pathways significantly overrepresented among the differential metabolites.

### 2.6 Cell Culture

All cell lines were cultured in Dulbecco’s Modified Eagle Medium (DMEM) (#11965092, Thermo Fisher Scientific, Waltham, MA, USA) supplemented with 10% fetal bovine serum (FBS) (#10099-141, Thermo Fisher Scientific, Waltham, MA, USA) and 1% penicillin-streptomycin solution (#15140122, Thermo Fisher Scientific, Waltham, MA, USA). Cells were maintained at 37°C in a humidified atmosphere containing 5% CO_2_, with the medium refreshed every 2–3 days. The human prostate cancer cell lines PC3 (Chinese Academy of Sciences, Beijing, China) and DU145 (Chinese Academy of Sciences, Beijing, China), along with the mouse prostate cancer cell lines RM-1 (Procell System, Wuhan, China) and TRAMP-C1 (Genetic Testing Biotechnology, Beijing, China), all tested negative for mycoplasma contamination as determined using a Mycoplasma Detection Kit (# D101, Vazyme, Nanjing, China). All cell lines used in this study were authenticated by Short Tandem Repeat (STR) profiling.

### 2.7 Identification and Characterization of Exosomal PD-L1

#### 2.7.1 Exosome Isolation from Human Plasma

After thawing, 1 mL plasma was filtered by a 0.8 µm filter (Millipore, SLAA025, Billerica, MA, USA), exosomes were collected by size exclusion chromatography (SEC), and the collected samples were poured into an ultrafiltration tube (Millipore, UFC801024, Billerica, MA, USA) for purification [44]. The samples were centrifuged at 4000× *g* for 15 min, yielding 200 µL of liquid.

#### 2.7.2 Electron Microscopy

After adding 10 µL of exosomes to the copper mesh, the mixture was incubated for ten minutes at room temperature. The samples were then washed with sterile distilled water, and any excess liquid was absorbed using filter paper. We pipetted 10 µL of 2% uranyl acetate dihydrate on the copper mesh for negative dyeing for 1 min, floating liquid was removed with filter paper, and dried under an incandescent lamp for 2 min. A HITACHI/H-7650 transmission electron microscope (Hitachi High-Technologies Corporation, Tokyo, Japan) was used to observe the copper mesh, and 80 kV was used for imaging.

#### 2.7.3 Nanoparticle Tracking Analysis

Nanoparticle tracking analysis was performed on exosome samples using a NanoSight LM10 system (NanoSight Ltd., Amesbury, UK), featuring a 405 nm laser and a high-sensitivity sCMOS camera (model Orca-Flash 2.8, Hamamatsu Photonics, Hamamatsu, Japan). All specimens were diluted 1000-fold in particle-free 1×PBS (pH 7.4) and introduced manually. The detection threshold was determined using ten parameters; both the automatic and minimum track lengths were adjusted to 10, with the minimum estimated particle size set at 10. Room temperature was documented by hand and kept below 25°C. Five 60-s videos were captured for each sample. Calibration procedures utilized polystyrene latex microspheres with diameters of 100, 200, and 400 nm.

#### 2.7.4 Western Blot

Total protein was extracted from cells and isolated exosomes using RIPA Lysis Buffer (# 89900, Thermo Fisher Scientific, Waltham, MA, USA) supplemented with Protease Inhibitor Cocktail (# 78429, Thermo Fisher Scientific, Waltham, MA, USA). The concentration of isolated exosomes and of cell lysates from human (PC3, DU145) and mouse (RM1, TRAMPC1) prostate cancer cell lines was quantified using the Pierce BCA Protein Assay Kit (#23225; Thermo Fisher Scientific, Waltham, MA, USA). A total of 20 µg of protein per sample was loaded onto 12% SDS-polyacrylamide gels. Protein specimens were resolved by 12% SDS-PAGE and then transferred onto polyvinylidene difluoride membranes (#IPVH00010; Merck Millipore, Billerica, MA, USA), followed by blocking with 5% non-fat milk solution. Following the incubation of the following primary antibodies overnight at 4°C, the membranes were incubated with the following: an anti-CD9 antibody (1:500; sc-13118; Santa Cruz Biotechnology, Dallas, TX, USA), an anti-TSG101 antibody (1:2500; ab125011; Abcam, Cambridge, UK), an anti-PD-L1 antibody (1:2500; ab213524; Abcam, Cambridge, UK), an anti-GAPDH antibody (1:1000; 2118; Cell Signaling Technology, Danvers, MA, USA), and an anti-β-Actin antibody (1:2000; 4967; Cell Signaling Technology, Danvers, MA, USA). The next day, primary antibodies were eliminated using TBST, and they were then incubated for one hour at room temperature with horseradish peroxidase (HRP)-conjugated secondary antibodies (Goat Anti-Rabbit IgG, 7074, 1:3000; Cell Signaling Technology, Danvers, MA, USA and Goat Anti-Mouse IgG, 7076, 1:3000; Cell Signaling Technology, Danvers, MA, USA). The enhanced chemiluminescence system (#23277; Thermo) was used to visualize the results. All Western blot experiments were performed in three independent biological replicates (n = 3).

#### 2.7.5 PD-L1 ELISA

The protein concentration was determined with a MicroBCA kit (Pierce, 23235, Waltham, MA, USA), the extracted exosomes were treated with 1% Triton X-100 sample lysate, and the PD-L1 was detected according to the standard operating procedure of the ELISA kit (AB214565, Abcam) instructions. The absorbance (OD value) of PD-L1 was detected by a multimode microplate reader (PerkinElmer EnSight, 450 nm; PerkinElmer, Waltham, MA, USA), and the actual concentration of PD-L1 was calculated based on the standard curve generated from the reference standards, using the equation: C = (A − *b*)/*m*, where C is the PD-L1 concentration, A is the absorbance, *b* is the y-intercept, and *m* is the slope of the standard curve.

#### 2.7.6 Mouse Models

All mice used in this study were treated humanely according to institutional animal care guidelines, and the study was approved by the Animal Ethics Committee of the Fifth Affiliated Hospital of Zhengzhou University (Ethics No:KY2022036).

The transgenic Adenocarcinoma of the Mouse Prostate (TRAMP) model, which is a well-established and widely used model for studying PC. Mice carrying this transgene develop PC spontaneously. The disease progression in these mice follows a predictable course, starting from prostatic intraepithelial neoplasia (PIN) to well-differentiated adenocarcinoma, and eventually to poorly differentiated and metastatic cancer [45].

A total of 16 male hemizygous C57BL/6 TRAMP mice (16 weeks old, with an average body weight of 25 g) were purchased from the Shanghai Model Organisms Center (Shanghai, China). In this work, mice were maintained until 20 weeks of age to allow for the development of metastases before subsequent experiments. The mice were randomly divided into three groups: a control group (saline, n = 8), a 16(R)-HETE-treated group (n = 4), and a 6-Keto-PGE1-treated group (n = 4). The compounds or vehicle were administered via drinking water. At 26 weeks of age, the control group was subsequently divided into two subgroups (n = 4) for treatment with either IgG or an anti-PD-L1 antibody. Mice in the 16(R)-HETE and 6-Keto-PGE1 intervention groups were treated with the anti-PD-L1 antibody. For two weeks, 200 µg of IgG or anti-PD-L1 monoclonal antibody were given to the mice twice a week. During the experiment, the mice were observed for their mental state, activity level, food and water intake, and aggression. The bedding was changed weekly, and the cages were washed and disinfected with alcohol to ensure the mice had adequate access to water and food at all times. At week 32, the mice were euthanized for further analysis.

### 2.8 Immunofluorescence and Hematoxylin and Eosin (HE) Staining

#### 2.8.1 Tissue Embedding and Sectioning

The prostate tissues from mice were fixed in 4% paraformaldehyde. The fixed tissues were then dehydrated using an automatic tissue processor (TP1020; Leica Biosystems, Nussloch, Germany). Following dehydration, the tissues were embedded in paraffin with the help of an embedding machine (EG1150; Leica Biosystems, Nussloch, Germany) and were allowed to cool. The paraffin-embedded tissue block was sectioned at a thickness of 4 µm using a microtome.

#### 2.8.2 Deparaffinization

For three hours, the tissue pieces were baked at 65°C in an oven. The sections were then deparaffinized by immersion in two changes of 100% xylene for 5 min each, followed by immersion in two changes of 100% ethanol, two changes of 95% ethanol, two changes of 80% ethanol, two changes of 65% ethanol, and two changes of 50% ethanol, each for 5 min. The sections were then rinsed with tap water for 5 min.

#### 2.8.3 HE Staining

Deparaffinized tissue sections were first stained with Mayer’s hematoxylin solution (ready-to-use concentration) for 4 min, followed by a 10-min rinse under running tap water. They were then differentiated in 70% ethanol containing 1% hydrochloric acid for 3 s, with a second 10-min tap water rinse performed afterward. Subsequent 1% eosin staining was carried out for 1 min, and the sections were dehydrated through a gradient of ethanol solutions (65%, 70%, 80%, 95%, two aliquots of absolute ethanol, two aliquots of xylene) with each step lasting 5 min. Finally, the sections were mounted using neutral balsam, allowed to air-dry completely, and subjected to microscopic imaging using an Axio Observer 3 microscope (Carl Zeiss AG, Jena, Germany).

### 2.9 T-Cell-Mediated Tumor Cell-Killing Assay

T cells were isolated from mouse spleens using the Pan T Cell Isolation Kit II (# 130-095-130, Miltenyi Biotec, Bergisch Gladbach, Germany) according to the manufacturer’s instructions. Briefly, spleens were aseptically removed and gently ground through a 70-μm cell strainer to obtain a single-cell suspension. Red blood cells were lysed using ACK Lysing Buffer (# A10492-01, Thermo Fisher Scientific, Waltham, MA, USA). T cells were then purified by negative selection. The isolated T cells were activated with anti-CD3 antibody (# 100302, 1:100; BioLegend, San Diego, CA, USA) and anti-CD28 antibody (# 102102, 1:100; BioLegend, San Diego, CA, USA) and anti-IL-2 (# 575402, 1:200; Biolegend, San Diego, CA, USA), and then treated with the indicated exosomes with or without anti-PD-L1 antibody (# BE0101, 1:200; Bio X Cell, West Lebanon, NH, USA) or IgG control (# BE0090, 1:200; Bio X Cell, West Lebanon, NH, USA) for 48 h. The T cells were subsequently co-cultured with RM1 and TRAMPC1 cells a 1:1 ratio, supplemented with CD3/CD28 antibody (100 and 20 ng/mL, respectively), IL-2 (10 ng/mL), along with the same exosomes and either anti-PD-L1 antibody or IgG control. After the co-culture for 5 days, the T cells were washed out with 1×PBS (pH 7.4) three times to remove T cells. 100% methanol was used to fix the surviving tumor cells, and a 0.5% crystal violet solution was used for staining the tumor cells. The dried plates were solubilized in 300 μL of 33% glacial acetic acid and shaken for 5 min. A 96-well plate was coated with 100 μL of the solution, which was then used to determine the absorbance at 570 nm with a SpectraMax iD5 Multi-Mode Microplate Reader (Molecular Devices, San Jose, CA, USA).

### 2.10 Transwell

A transwell assay was utilized to evaluate cell migration ability. Briefly, human prostate cancer cell lines PC3 and DU145 were re-suspended in serum-depleted medium and plated into the upper compartment of transwell inserts (8-μm pore diameter, Corning, Corning, NY, USA) at a seeding density of 2 × 10^4^ cells per well. The lower chamber was supplemented with medium containing 10% fetal bovine serum (FBS) (# 10099-141, Thermo Fisher Scientific, Waltham, MA, USA) to serve as a chemoattractant. Following 48 h of incubation at 37°C, non-migratory cells adhering to the upper surface of the membrane were wiped off using a cotton swab. Migratory cells on the lower membrane surface were fixed with 4% paraformaldehyde for 15 min and stained with 0.1% crystal violet solution for 10 min. Stained cells were observed and counted under a light microscope (Axio Observer 3, Carl Zeiss, Jena, Germany) at 200× magnification. The number of migrated cells was quantified and presented as the mean ± standard deviation (SD) from three replicate wells.

### 2.11 Colony Formation

A clonogenic assay combined with crystal violet staining was employed to evaluate cell proliferation. Briefly, 250 PC3 and DU145 cells were plated into each well of a 6-well culture plate and maintained in complete medium for 14 days of incubation. The cells were subsequently rinsed twice with 1×PBS (pH 7.4), fixed in 4% paraformaldehyde for 15 min, and stained with 0.5% crystal violet solution at room temperature for 30 min. Following thorough washing with distilled water, the plates were allowed to air-dry, and colonies consisting of over 50 cells were counted under a light microscope. The stained colonies were visualized and quantitatively analyzed using ImageJ software (Version 1.53t; NIH, Bethesda, MD, USA). The colony count was presented as the mean ± SD derived from three replicate wells.

### 2.12 Wound Healing

The cell scratch assay was performed to measure cell migration. PC3 and DU145 cells were seeded in 6-well plates at a density of 2 × 10^5^ cells per well and cultured until they reached confluence. A sterile 200-μL pipette tip was used to create a straight scratch in the cell monolayer. Subsequently, 1×PBS (pH 7.4) was used to wash the plates in order to get rid of the detached cells and debris. The cells were cultivated in serum-free medium enriched with 1% FBS at 37°C for 24 h, during which the wound healing process was monitored and documented.

### 2.13 Data Analysis

Differential expressions of metabolites were identified by intersecting the results of the Wilcoxon rank sum test, performed using the ‘wilcox.test’ function in the ‘Stats’ R package (V.4.2.0). The results of Partial Least Squares Discriminant Analysis (PLS-DA) were performed using the ‘plsda’ function in the mixOmics R package (V.6.20.0) [46]. The Variable Importance in Projection (VIP) scores for the PLS-DA were calculated using the ‘PLSDA.VIP’ function in the ‘RVAideMemoire’ R package (V.0.9-83), with a cutoff value set at VIP > 1. KEGG pathway enrichment analysis was performed using the ‘enricher’ function in the ‘clusterProfiler’ R package (V.4.9.0.002) with default parameters [47]. The KEGG background metabolite set was constructed from all metabolites detected in this study that could be annotated to KEGG.

Correlations between metabolites and exosomal PD-L1 were evaluated using the ‘cor.test’ function in the ‘Stats’ R package (V.4.2.0), with the ‘method’ parameter set to Pearson. For the correlation between PD-L1-related gut metabolites and gut microbiota, the ‘cor.test’ function was used, while with the ‘method’ parameter set to Spearman. All statistical analyses were performed using R software (version 4.2.0).

Differences between groups were assessed using the Wilcoxon rank sum test due to continuous variables. A significance level of *p* < 0.05 was considered statistically significant. Continuous variables with non-normal distribution are presented as median (range).

## 3 Results

### 3.1 Gut Metabolites Associated with Plasma Exosomal PD-L1 in PC

**Figure 1:**
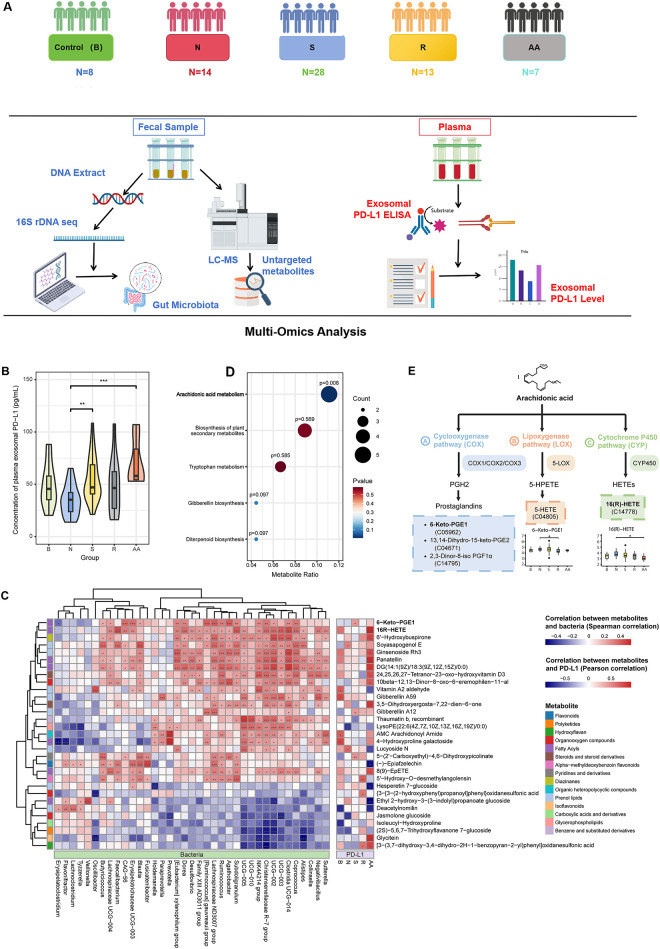
Gut microbiota-related metabolites 16(R)-HETE and 6-Keto-PGE1 were found to be associated with exosomal PD-L1 levels. (**A**) The study design of this work. (**B**) Comparisons of plasma exosomal PD-L1 concentrations among different patient groups. (**C**) KEGG pathway enrichment analysis based on differential metabolites identified between various group pairs (Group B vs. N; N vs. S; N vs. R; N vs. AA; S vs. R; and R vs. AA). Only the “Arachidonic acid metabolism (AA)” pathway showed significant enrichment (*p* = 0.0079). (**D**) Correlations between metabolites and exosomal PD-L1 were calculated using Pearson correlation, while correlations between PD-L1-related gut metabolites and gut microbiota were conducted using Spearman correlation. (**E**) Location of the five differential metabolites enriched in the AA pathway. 6-Keto-PGE1 is a downstream metabolite of the Prostaglandins produced in the AA metabolic pathway. 16(R)-HETE is produced through the metabolism of arachidonic acid (AA) by cytochrome P450 (CYP) enzymes. The Wilcoxon rank-sum test was used to compare continuous variables between groups. *p < 0.05; **p < 0.01; ***p < 0.001

To identify the main features of the gut microbiome correlated with PD-L1 levels in PC patients, we recruited a total of 70 patients. Among them, the control groups included treatment-naïve patients with varying disease severity, including benign prostatic hyperplasia (group B, n = 8) and treatment-naïve PC patients (group N, n = 14). Medication-receiving patients were treated with different drugs with different susceptibilities, including 28 hormone-sensitive (group S), 13 castration-resistant (group R), and 7 castration-resistant with abiraterone acetate (group AA) PC patients. Multi-omics data were collected and analyzed from each group, including metabolomics and metagenomics (Fig. 1A).

Patients in these groups showed no significant differences in age, body mass index (BMI), or alkaline phosphatase (AKP) (Table 1). AKP is a key biomarker in PC, indicating bone metastasis, monitoring disease progression, and evaluating treatment response. Serum prostate-specific antigen (PSA) is a known biomarker for PC diagnosis and management, and its levels were significantly increased in group N as compared with those in group B. In parallel, PSA level was significantly decreased in group S, while significantly increased in groups R and AA. These results confirmed that PSA levels could indicate different stages and treatment outcomes of PC.

We also investigated the association of plasma exosomal PD-L1 level with PC, gut bacteria, and fecal metabolites. The transmission electron microscopy (TEM) and ELISA were used for detecting and validating exosomal PD-L1. The TEM results showed the characteristic morphology of exosomes, including particle size distribution and surface protein markers (Supplementary Fig. S1). Notably, patients in group B and those receiving medication (Groups S, R, and AA) showed elevated plasma exosomal PD-L1 levels compared to untreated PC patients. Although Groups B and R did not show statistically significant differences, they showed a trend toward higher PD-L1 levels than Group N (Fig. 1B).

To identify critical metabolites and associated biological pathways, a comparative analysis of metabolic profiles across experimental groups was performed, followed by enrichment evaluation using the KEGG database. We found significant enrichment only in the “Arachidonic acid metabolism (AA)” pathway (*p* = 0.0079, Fig. 1C). Among the five metabolites associated with this pathway, 16(R)-HETE and 6-Keto-PGE1 were positively correlated with PD-L1 levels in groups AA and S, respectively. Moreover, a positive correlation was observed between these metabolic compounds and the prevalence of multiple bacterial genera known to synthesize short-chain fatty acids (SCFAs), such as Coprococcus, Ruminococcus, Subdoligranulum, Blautia, Faecalibacterium, and Clostridium (Fig. 1D). SCFAs produced by these bacteria can modulate the expression and activity of enzymes involved in AA metabolism, such as COX, LOX, and CYP enzymes, thus impacting the production of AA metabolites 16(R)-HETE and 6-Keto-PGE1 (Fig. 1E).

### 3.2 16(R)-HETE and 6-Keto-PGE1 Promote PD-L1 in PC Cells

**Figure 2:**
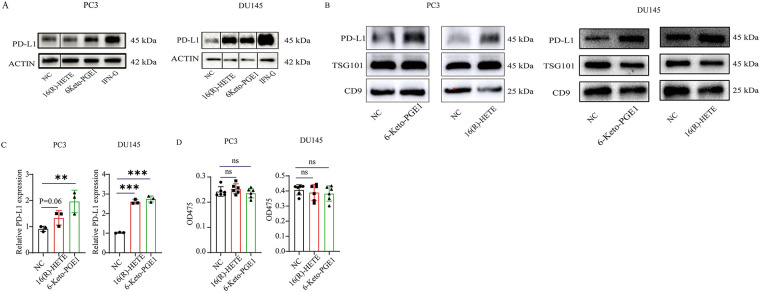

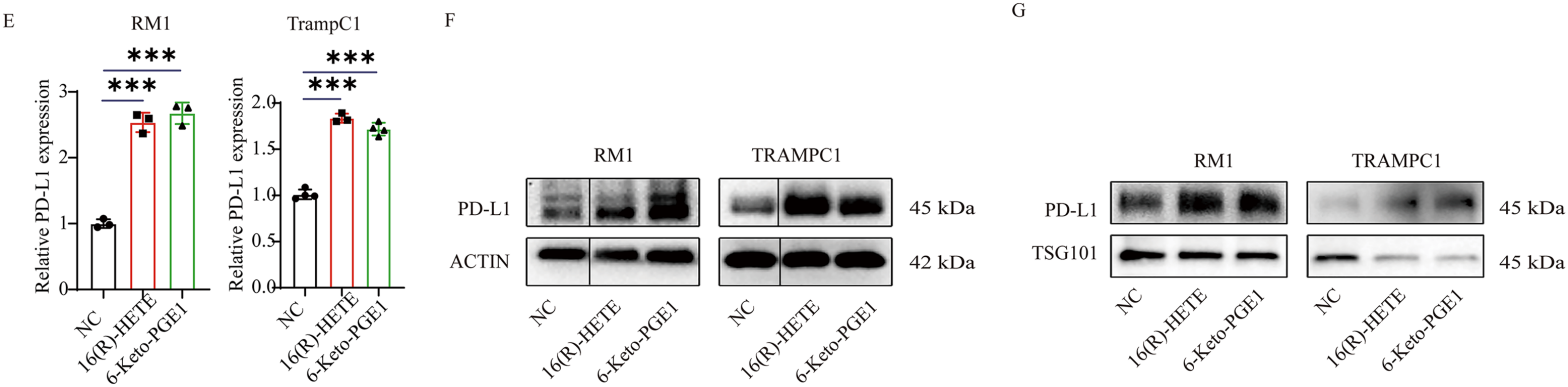
Effects of 16(R)-HETE and 6-Keto-PGE1 on PD-L1 expression and cell viability in human and mouse prostate cancer cell lines. (**A**) PD-L1 protein expression in human prostate cancer cell lysates treated with 16(R)-HETE and 6-Keto-PGE1. Two human PC cell lines, PC3 and DU145, were used. (**B**) Exosome PD-L1 protein expression in the two human PC cell lines treated with 16(R)-HETE and 6-Keto-PGE1. (**C**) The mRNA expression of PD-L1 in human prostate cancer cells treated with 16(R)-HETE and 6-Keto-PGE1. (**D**) Cell viability of prostate cancer cell line PC3 and DU145 treated with 16(R)-HETE and 6-Keto-PGE1. (**E**) The mRNA expression of PD-L1 in mouse prostate cancer cells treated with 16(R)-HETE and 6-Keto-PGE1. Two mouse PC cell lines, namely RM1 and TRAMPC1, were used. (**F**) PD-L1 protein expression of mouse prostate cancer cell lysates treated with 16(R)-HETE and 6-Keto-PGE1. (**G**) Exosome PD-L1 protein expression of mouse prostate cancer cells treated with 16R-HETE and 6-Keto-PGE1. ^ns^*p ≥* 0.05; **p < 0.01; ***p < 0.001

We focused on the two metabolites, 16(R)-HETE and 6-Keto-PGE1, and validated their effects on PD-L1 expression in both human and mouse PC cell lines. In two human PC cell lines, PC3 and DU145, we observed that treatment with 0.1 μg/mL 6-Keto-PGE1 could significantly up-regulate PD-L1 protein levels, while 0.1 μg/mL 16(R)-HETE could significantly up-regulate PD-L1 protein levels in DU145 (Fig. 2A), which was also found in exosomes derived from these cells (Fig. 2B). In parallel, the mRNA expression levels of PD-L1 were significantly altered in response to these treatments (Fig. 2C). These results together indicated that the treatments influenced PD-L1 expression through both intracellular and extracellular mechanisms, and at both mRNA and protein levels. Furthermore, we found that treatment with 16(R)-HETE and 6-Keto-PGE1 alone did not significantly reduce the viability of PC3 and DU145 cells, indicating that 16(R)-HETE and 6-Keto-PGE1 treatments increased PD-L1 expression in human PC cell lines while not promoting cell proliferation (Figs. 2D and S2).

We also validated our findings using two mouse PC cells, namely RM1 and TRAMPC1. Consistent with the results from human cells, we found that both 16(R)-HETE and 6-Keto-PGE1 treatments led to an up-regulation of PD-L1 protein levels in mouse cell lysates (Fig. 2E). This up-regulation was also observed in exosomes derived from mouse PC cells, indicating a conserved mechanism across species (Fig. 2F,G). Moreover, the treatments induced significant changes in PD-L1 mRNA expression in the mouse cells, mirroring the transcriptional regulation seen in human cells.

### 3.3 The Effect of 16(R)-HETE and 6-Keto-PGE1 on PD-L1 Expression in a Subcutaneous Mice Model

**Figure 3:**
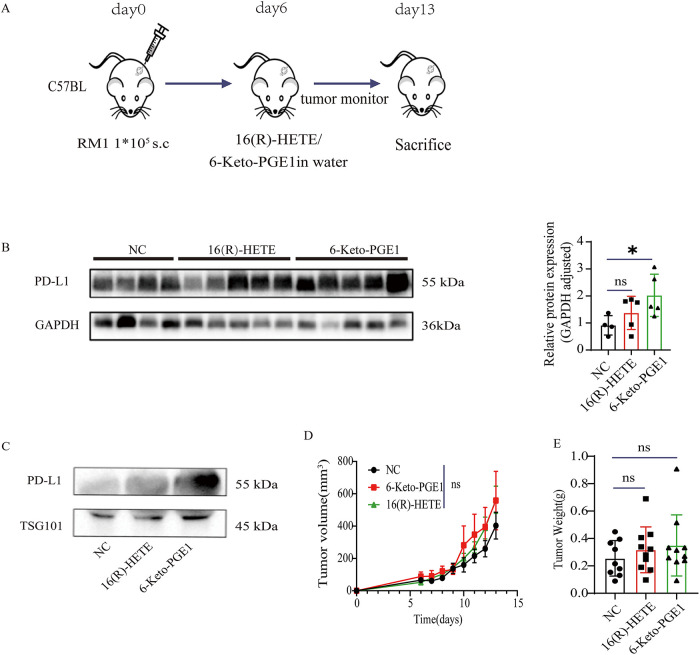
Impact of 16(R)-HETE and 6-Keto-PGE1 on Tumor Growth and PD-L1 Expression in Mouse Prostate Cancer. (**A**) Scheme of subcutaneous RM1 mouse prostate cancer models. (**B**) PD-L1 protein expression of subcutaneous RM1 tumor tissues treated with 16(R)-HETE and 6-Keto-PGE1. (**C**) Plasma exsomal PD-L1 protein expression of subcutaneous RM1 tumor models treated with 16(R)-HETE and 6-Keto-PGE1. (**D**,**E**) Tumor growth curve and weight. ^ns^*p ≥* 0.05; *p < 0.05

**Figure 4:**
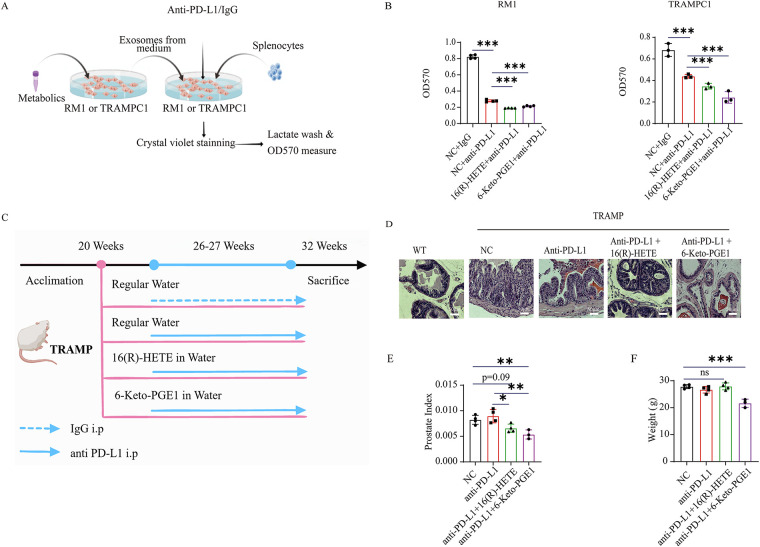
Gut microbiota-related metabolite 16(R)-HETE and 6-Keto-PGE1 enhance the anti-PD-L1 efficacy of mouse PC cell lines and TRAMP models. (**A**) Scheme of *in vitro* killing assay experiment. (**B**) *In vitro* killing assay of mouse splenocytes on RM1 and TRAMPC1 cells. (**C**) Scheme of mouse TRAMP prostate cancer model pre-treated with 16(R)-HETE and 6-Keto-PGE1 and combined with anti-PDL1 therapy. (**D**) HE staining (400×) of prostate TRAMP mouse treated with 16(R)-HETE, 6-Keto-PGE1 in combination with IgG or anti-PD-L1. (**E**) Prostate Index of TRAMP mouse treated with 16(R)-HETE, 6-Keto-PGE1 in combination with IgG or anti-PD-L1. (**F**) Body weight of TRAMP mice treated with 16(R)-HETE, 6-Keto-PGE1 in combination with IgG or anti-PD-L1. ^ns^*p ≥* 0.05; *p < 0.05; **p < 0.01; ***p < 0.001

We then evaluated the effects of 16(R)-HETE and 6-Keto-PGE1 on tumor growth using subcutaneous RM1 PC mouse models (Fig. 3A). When treated with either of the metabolites, we found a significantly higher PD-L1 protein level in both the tumor tissues (Fig. 3B,C) and plasma exosomes (Fig. 3D) following treatment with these metabolites. Additionally, there was no significant effect of 16(R)-HETE or 6-Keto-PGE1 on tumor growth compared to control groups (*p* > 0.05, *t*-test; Fig. 4D,E). These results were in line with our observations in the cell lines.

### 3.4 The Effect of 16(R)-HETE and 6-Keto-PGE1 on Anti-PD-L1 Efficacy in PC Cell Lines and Mouse Models

To evaluate the enhancement of 16(R)-HETE and 6-Keto-PGE1 on the efficacy of anti-PD-L1 therapy in PC treatment, we conducted an *in vitro* investigation on mouse PC cell lines RM1 and TRAMPC1 (Fig. 4A). Due to the complexity of constructing human models and the need for a matching major histocompatibility complex (MHC) or other specific models to target or eliminate certain cells or organisms effectively, we only conducted the killing assay on mouse cell lines. We found that both metabolites enhanced the immune cell-mediated cytotoxicity of mouse splenocytes against RM1 and TRAMPC1 cells than the PD-L1 antibody treatment alone (*p* < 0.05, *t*-test; Fig. 4B). We further validated these findings *in vivo* using the TRAMP mouse model (Fig. 4C). Briefly, we treated TRAMP mice and control groups with a combination of metabolites and anti-PD-L1 antibodies. Upon histological examination, prostate tissues obtained from the experimental mice showed markedly decreased tumor burden in subjects administered the combination therapy relative to the group receiving IgG monotherapy [48] (Fig. 4D). This observation was further supported by the Prostate Index measurements, which showed a significant decrease in tumor burden in the combination therapy group (Fig. 4E). We also measured the body weight of the TRAMP mice throughout the treatment period to assess potential systemic toxicity (Fig. 4F). The body weights of the mice remained stable across all treatment groups, indicating that the administration of 16(R)-HETE, 6-Keto-PGE1, and anti-PD-L1 did not result in adverse systemic effects.

## 4 Discussion

Due to the aggressive nature and resistance to conventional therapies [7], treating PC is challenging and calls for non-canonical treatment strategies. Here, we leveraged a multi-omics research on a PC cohort and identified two gut microbiota-associated metabolites, namely 16(R)-HETE and 6-Keto-PGE1, that could enhance the efficacy of PD-L1 blockade treatment via increasing the PD-L1 expression at both mRNA and protein levels. We validated the effects of the combined treatment using two human and mouse cell lines for each and a TRAMP model, providing a promising strategy for further application and enhancement of immune checkpoint blockade (ICB) therapies in PC.

The association of PD-L1 expression with cancer development involves multifaceted interactions [34,40]. While PD-L1 is typically associated with immune evasion by tumors, its role in promoting or inhibiting tumor growth is not straightforward [34]. Several studies have indicated that high PD-L1 expression can correlate with poor prognosis in various cancers, as it allows tumors to escape immune surveillance [30,36,39,40,49]. However, other studies suggest that PD-L1 expression can also indicate an active immune response within the tumor microenvironment, potentially enhancing the effectiveness of ICB therapies [50]. Previous studies have shown that gut microbes and fecal metabolites exert a profound impact on the peripheral immune system, including the efficacy of anti-PD-1/PD-L1 therapy [34,50]. For the first time, we found that plasma exosomal PD-L1 in PC patients correlated with *Ruminococcus gauvreauii* group and Flavonifractor, belonging to Lachnospiraceae and Ruminococcaceae, respectively, which are positively associated with anti-PD1/PD-L1 immunotherapy response.

Metabolites derived from arachidonic acid, such as prostaglandins and hydroxyeicosatetraenoic acids (HETEs), have been implicated in cancer progression and immune modulation. Specifically, 6-Keto-PGE1 and 16(R)-HETE are bioactive lipid mediators that have been confirmed to have various biological activities in different cancer types [51,52]. However, their roles in modulating PD-L1 expression in PC cells and their potential impact on the efficacy of anti-PD-L1 therapies remain poorly understood. In this work, 16(R)-HETE and 6-Keto-PGE1 were observed to promote the overall level of PD-L1 in the model without impacting tumor growth. 6-Keto-PGE1 performs a more pronounced effect, suggesting a potential regulatory role of these metabolites in the immune evasion mechanisms of PC cells mediated through PD-L1 expression. The superior efficacy of 6-Keto-PGE1 in upregulating PD-L1 underscores the importance of understanding the distinct biochemical pathways through which these metabolites exert their effects.

In the context of anti-PD-L1 treatment, our study revealed that 16(R)-HETE was more effective in inhibiting PC tumor weight than 6-Keto-PGE1. This intriguing result suggests that while 6-Keto-PGE1 may be more efficient at upregulating PD-L1, 16(R)-HETE might interact with additional pathways or mechanisms that enhance the efficacy of anti-PD-L1 therapy. It is plausible that 16(R)-HETE affects the tumor microenvironment or immune cell infiltration in a manner that synergizes with PD-L1 blockade, thereby leading to more substantial tumor weight reduction. Therefore, their role in tumor biology may be more nuanced, potentially involving complex interactions with the tumor microenvironment and immune system. Additionally, aging modulates gut microbiota composition, which in turn alters metabolite profiles. Relevant studies indicate that aging reduces beneficial bacteria and elevates pro-inflammatory metabolites [53,54]. It is plausible that such age-related changes in gut microbiota and metabolites may also impact the abundance of the key metabolites identified in our study, though this remains to be verified in future research. If this association exists, it could further affect anti-PD-L1 efficacy by regulating PD-L1 expression or the immune microenvironment.

Notably, we employed a primary tumor model for this study, which offers several advantages, including the ability to closely mimic the tumor microenvironment and the progression of human PC. This system enables a more precise evaluation of treatment effectiveness and immunological dynamics in the tumor microenvironment. Collectively, the integrated *in vitro* and *in vivo* results indicate that the gut microbiome-derived metabolites 16(R)-HETE and 6-Keto-PGE1 substantially potentiate the anti-tumor activity of PD-L1 blockade in prostate cancer, presenting a viable strategy to advance clinical management of this disease.

## 5 Limitations of the Study

Our results are not without limitations. The primary constraint arises from the limited cohort size, which may affect the broader applicability of the conclusions. Future investigations involving expanded sample sets would help to strengthen and refine these observations. Moreover, the potential risks associated with the elevation of PD-L1 levels were not comprehensively addressed within the scope of this research; further investigation is required to elucidate the safety profile of PD-L1 modulation. While recent studies underscore the capacity of intestinal flora to influence antitumor immunological reactions during therapeutic interventions [23–25,55], the specific bacterial strains implicated in the observed effects were not identified, highlighting the need for future studies to isolate and characterize these microbes for more precise insights. In addition, the underlying mechanisms driving our observations remain inadequately explained, thereby calling for additional research to delineate the involved molecular pathways and interactions.

## Supplementary Materials





## Data Availability

Lead contact: Further information and requests for resources and reagents should be directed to and will be fulfilled by the lead contact, Yang Mi (yangmi198@zzu.edu.cn). Materials availability: This study did not generate new reagents. Data and code availability: The data that support the findings of this study are available from the corresponding author upon reasonable request. The Illumina MiSeq raw sequencing reads were deposited into the NCBI Sequence Read Archive (SRA) database (Accession Number: PRJNA703725). The complete list of differential metabolites is provided in Supplementary Table S1.

## Supplementary Materials

The supplementary material is available online at https://www.techscience.com/doi/10.32604/or.2025.072661/s1.
