# Supplementary material for: Gut Associated Metabolites Enhance PD-L1 Blockade Efficacy in Prostate Cancer
Source: Oncol Res. 2026 Jan 19;34(2):23. doi: 10.32604/or.2025.072661 (PMC12848745; doi:10.32604/or.2025.072661)
Supplement: Supplementary file 1 [file OncolRes-34-72661-s001.docx]

**Supplementary Figure S1.** The transmission electron microscopy of exosomes, including particle size distribution and surface protein markers.

**Supplementary Figure S2.** Gut microbiota-related metabolite 16(R)-HETE and 6-Keto-PGE1 enhance the anti-PD-L1 efficacy *in vitro*. (A) Colony formation efficacy of PC3 and DU145 cells treated with 16(R)-HETE or 6-Keto-PGE1. (B) Transwell assay of PC3 and DU145 cells treated with 16(R)-HETE or 6-Keto-PGE1. (C) Wound healing ability of PC3 and DU145 cells treated with 16(R)-HETE or 6-Keto-PGE1. (D) RM1 and TrampC1 cells are alive after the *in vitro* killing assay.
